# Serum metabolome and liver transcriptome reveal acrolein inhalation-induced sex-specific homeostatic dysfunction

**DOI:** 10.1038/s41598-023-48413-w

**Published:** 2023-12-01

**Authors:** Devin I. Alewel, Katherine M. Rentschler, Thomas W. Jackson, Mette C. Schladweiler, Anna Astriab-Fisher, Paul A. Evansky, Urmila P. Kodavanti

**Affiliations:** 1 Oak Ridge Institute for Science and Education Research Participation Program, U.S. Environmental Protection Agency, Research Triangle Park, NC USA; 2 Public Health and Integrated Toxicology Division, Center for Public Health and Environmental Assessment, U.S. Environmental Protection Agency, 109 T.W. Alexander Dr., Research Triangle Park, NC 27711 USA

**Keywords:** Transcriptomics, Metabolomics, Neuroendocrinology, Endocrine system and metabolic diseases, Environmental sciences

## Abstract

Acrolein, a respiratory irritant, induces systemic neuroendocrine stress. However, peripheral metabolic effects have not been examined. Male and female WKY rats were exposed to air (0 ppm) or acrolein (3.16 ppm) for 4 h, followed by immediate serum and liver tissue collection. Serum metabolomics in both sexes and liver transcriptomics in males were evaluated to characterize the systemic metabolic response. Of 887 identified metabolites, > 400 differed between sexes at baseline. An acrolein biomarker, 3-hydroxypropyl mercapturic acid, increased 18-fold in males and 33-fold in females, indicating greater metabolic detoxification in females than males. Acrolein exposure changed 174 metabolites in males but only 50 in females. Metabolic process assessment identified higher circulating free-fatty acids, glycerols, and other lipids in male but not female rats exposed to acrolein. In males, acrolein also increased branched-chain amino acids, which was linked with metabolites of nitrogen imbalance within the gut microbiome. The contribution of neuroendocrine stress was evident by increased corticosterone in males but not females. Male liver transcriptomics revealed acrolein-induced over-representation of lipid and protein metabolic processes, and pathway alterations including Sirtuin, insulin-receptor, acute-phase, and glucocorticoid signaling. In sum, acute acrolein inhalation resulted in sex-specific serum metabolomic and liver transcriptomic derangement, which may have connections to chronic metabolic-related diseases.

## Introduction

Metabolic disease prevalence is increasing worldwide, creating a major public health challenge^1,2^. According to 2014 World Health Organization estimates, over 422 million people live with diabetes^3^, which is expected to increase 25% by 2030^4^. Traditional risk factors for metabolic disease include a sedentary lifestyle, high-fat diet, aging, genetics, and hormonal imbalance^5^. However, environmental air pollutant exposures have also been associated with increased incidences of metabolic diseases in humans^6,7^. Air pollutants have been associated with an increased risk of type 2 diabetes mellitus (T2DM), insulin resistance, dyslipidemia, and non-alcoholic fatty liver disease (NAFLD)^8–10^. Thus, more research is needed to understand the complex interactive effects of prevalent air pollutants on metabolic processes, especially in those with pre-existing conditions or susceptible populations.

Hallmarks of metabolic disorders often include insulin resistance, adipose-alterations, and an amalgamation of inflammatory mediators (pro-inflammatory cytokines)^11^. Various mechanistic pathways link respiratory injury with systemic inflammation and stress, which is postulated to provide an indirect route for inhaled pollutant-induced metabolic abnormalities^10,12–14^. Recently, hyperactivation of the sympathetic-adrenal-medullary (SAM) and hypothalamic–pituitary–adrenal (HPA) axes have been identified, whereby autonomic responses to inhaled pollutants modulate inflammatory and cellular signaling in adipose tissues, muscle, and liver^15–19^. Inhaled pollutants, such as ozone and particulate matter (PM), increase circulating adrenal catecholamines and glucocorticoids, which, through their extensive and tightly regulated cellular receptor dynamics, produce homeostatic alterations in metabolic and immune processes to counter stress effects^20,21^. Acute ozone exposure in rats is associated with a broad derangement of lipid and amino acid metabolic processes, as well as glucocorticoid-dependent effects on insulin-signaling in liver and brown and white adipose tissue^15,22^. However, systemic metabolic outcomes for other structurally distinct high priority irritant air pollutants have not been thoroughly investigated.

Acrolein is an environmentally ubiquitous hazardous air pollutant regulated by the United States Environmental Protection Agency^23^. Acrolein is a volatile aldehyde abundantly released from wildfires, industrial activities, and automobile exhaust. Human exposure is commonly associated with cigarette smoking, with acrolein being the most hazardous non-carcinogenic chemical in tobacco smoke^24,25^. As a strong electrophile, acute acrolein inhalation causes airway epithelial protein adduct formation, oxidative stress, and mitochondrial dysfunction, and repeated long-term exposure is associated with exacerbation of respiratory illnesses including asthma and chronic obstructive pulmonary disease^26^. Although impacts of this sensory irritant are well characterized in the respiratory tract, systemic effects are poorly understood. In rodents, acrolein physicochemically reacts in nasal cavities, which may stimulate trigeminal nerve endings, providing a potential route to signal systemic stress reactions^27^. Previous rat studies have demonstrated that acute acrolein exposure induces cardiovascular dysfunction and glucose intolerance^28,29^. Recently, we found that exposure to 3.16 ppm acrolein in Wistar-Kyoto (WKY) rats activates SAM and HPA axes^30^. However, metabolic outcomes associated with acrolein remain under-investigated, although some epidemiological evidence has linked long-term acrolein exposure with metabolic abnormalities in humans. In a 2016 National Health and Nutrition Examination Survey, individuals with higher urine acrolein-specific metabolite 3-hydroxypropyl mercapturic acid (3-HPMA) were positively associated with diabetes and insulin resistance phenotypes^31^. More recent epidemiological assessments suggest adults exposed to acrolein may have an increased risk for dyslipidemia, impaired glycemic control, and T2DM^32,33^. Still, the causal evidence for inhaled acrolein-induced systemic metabolic effects remains limited^29^.

The goal of the current study was to utilize global serum metabolomic and liver transcriptomic approaches to gain insight into the homeostatic metabolic effects and potential mechanisms of acute acrolein inhalation-induced metabolic dysfunction. Serum metabolite profiling was used to detect phenotypic changes in homeostatic disruption, whereby unique biochemicals may be associated with specific stress-dependent alterations. Due to the previously reported sex-specific nature of acrolein to drive neuroendocrine activation and markers of systemic stress in male rats^30^, we performed mRNA sequencing in male liver tissue. We exposed male and female WKY rats nose-only to air or acrolein for 4 h and then immediately collected samples for serum metabolomic and liver transcriptomic assessments, hypothesizing acute exposure would be associated with metabolic dysregulation in male but not female rats, which would be supported by concordant liver transcriptomic changes in males.

## Results

### Acrolein exposure alters clinical markers of metabolic stress in males

To determine if acrolein-induced effects are evident in clinical metabolic markers, levels of blood glucose and total serum free fatty acids (FFA) were assessed. Changes in circulating glucose and clinical chemistry markers have been shown to be sensitive indicators of systemic metabolic stress following ozone inhalation^19,34^. Assessment of blood glucose and serum FFA indicated higher levels in acrolein-exposed male but not female rats when compared to air controls (Supplemental Fig. 1A and B).

### Acute acrolein exposure induces changes in the serum metabolome and increases serum 3-HPMA, a biomarker of exposure

**Figure 1:**
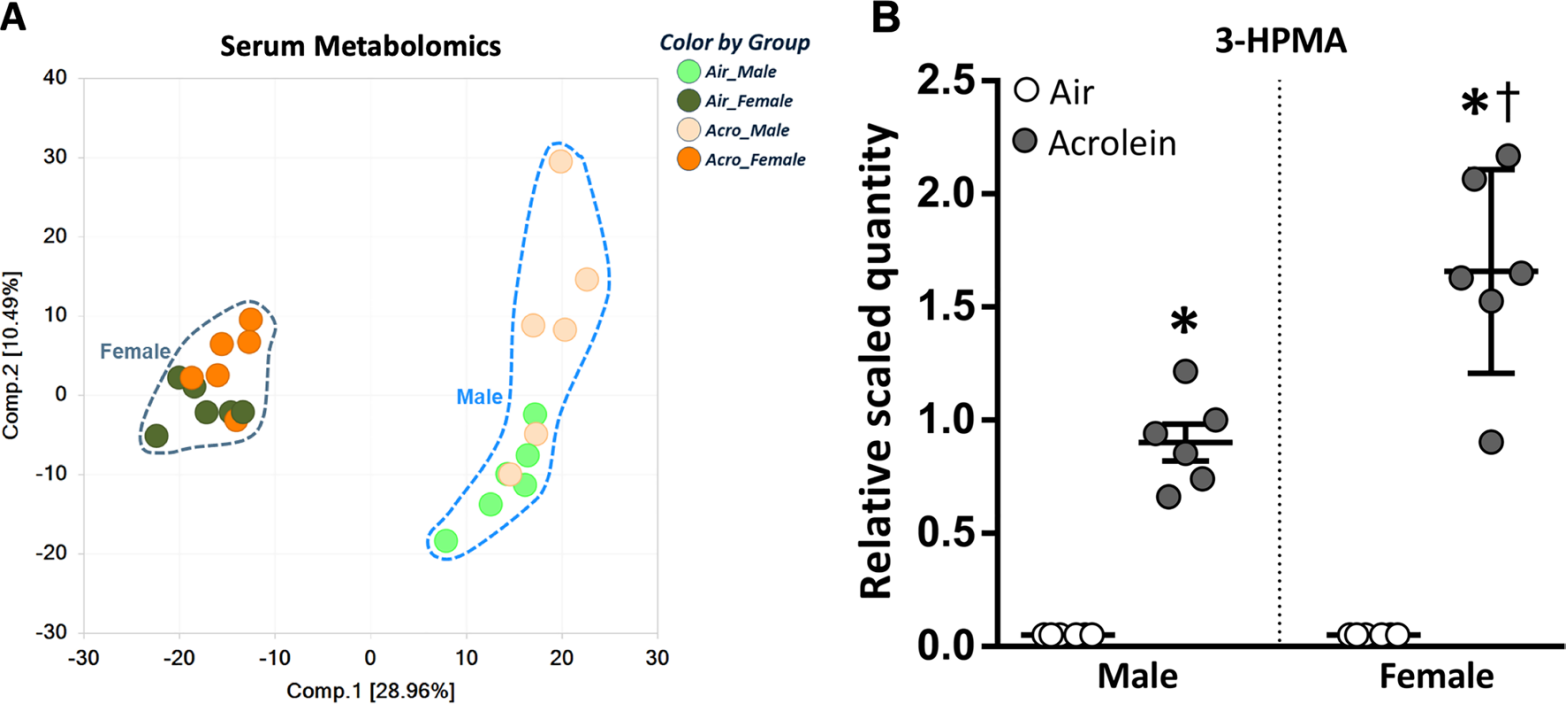
Principal component analysis depicting global metabolite changes in males and females exposed to air or acrolein and effect of acrolein on serum biomarker 3-hydroxypropyl mercapturic acid (3-HPMA). All serum samples for males and females are depicted in (**A**) (n = 6/group/sex). Acrolein-induced changes in the serum levels (relative scaled quantity) of metabolite 3-HPMA are depicted in (**B**) for male and female rats (n = 6/group/sex). *Significant acrolein effect (*p* $\le$ 0.05); †Significant sex difference (*p* $\le$ 0.05).

A total of 887 known biochemicals were identified through serum metabolomic assessment across amino acid, peptide, carbohydrate, energy, lipid, and nucleotide super-pathways (Supplemental Table 1). A summary of quantified statistically significant (*p* $\le$ 0.05) or near significant (*p* = 0.05–0.10) biochemicals are shown in Table 1. A total of 174 metabolites were significantly changed in males exposed to inhaled acrolein, whereas females only exhibited 50 metabolite alterations (*p* $\le$ 0.05). Between air-exposed male and female rats, nearly half (419) of quantified metabolites differed at baseline (*p* $\le$ 0.05) (Table 1). Air-exposed females had generally higher levels of circulating tyrosine, tryptophan, and steroid metabolites, but lower levels of branched-chain amino acids (BCAA), short- and medium-chain fatty acids, acyl carnitines, mono- and di-hydroxy fatty acids, and endocannabinoids compared to air-exposed males. Principal component analysis (PCA) of serum metabolic profiles revealed clear segregation between male and female rats (Fig. 1A). Assessed separately through PCA, female air and acrolein samples overlapped, appearing metabolically similar (Supplemental Fig. 2A). However, there was a stronger separation by exposure in males (Supplemental Fig. 2B). The biochemical most affected by acrolein inhalation in both male and female rats was the prominent metabolite of acrolein, 3-HPMA (Fig. 1B). Serum 3-HPMA levels were 18-fold higher in acrolein-exposed males and 33-fold higher in acrolein-exposed females when compared to sex-matched air controls (Fig. 1B).

**Table 1:**
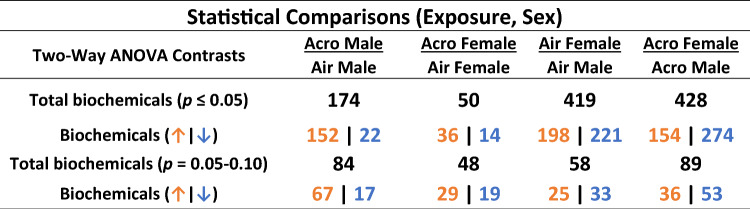
Summary of significantly changed metabolites in serum at *p* $\le$ 0.05 and *p* = 0.05–0.10 for male and female air- or acrolein-exposed rats (n = 6/group/sex).

Serum metabolomics were performed in male and female Wistar-Kyoto rats exposed to air (0 ppm) or acrolein (~ 3.16 ppm) for 4 h. Two-way analysis of variance results shows the number of significantly increased (orange) or decreased (blue) metabolite levels across comparisons.

### Acrolein-induced changes in steroid metabolites show sex differences in steroidogenesis involving corticosterone and 20α-dihydroprogesterone

**Figure 2:**
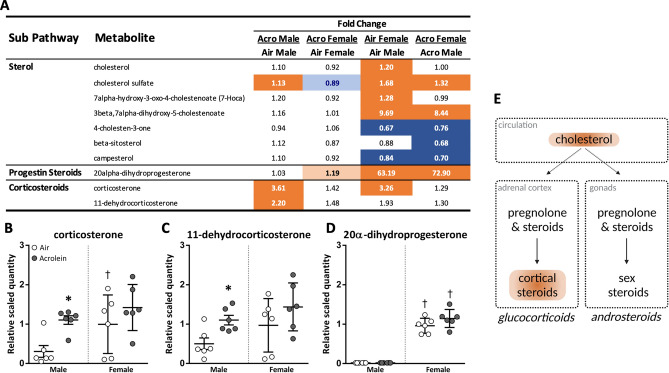
Acrolein-induced changes in steroid metabolism of male and female rats. (**A**) depicts a heatmap table of steroid metabolites expressed as fold change (relative quantity). Dark orange and dark blue are *p* $\le$ 0.05; light orange and light blue are *p* = 0.05–0.10. Serum steroidogenic metabolites corticosterone (**B**), 11-dehydrocorticosterone (**C**), and 20α-dihydroprogesterone (**D**) were identified in serum metabolomic assessment of air- and acrolein-exposed rats (shown as relative scaled quantity, scatter dot plots show all points; mean ± SEM, *n* = 6/group/sex). *Significant acrolein effect (*p* $\le$ 0.05); †Significant sex difference (*p* $\le$ 0.05). Schema of adrenal steroidogenesis is also shown, with points of acrolein impact highlighted (**E**).

Global metabolite assessment indicated acrolein sex-specifically altered steroid biosynthesis metabolites (Fig. 2). The steroid precursor cholesterol sulfate was significantly higher in acrolein-exposed males when compared to air group. However, acrolein did not impact this metabolite in females, although female rats had higher basal cholesterol and cholesterol sulfate levels compared to males (Fig. 2A). Serum corticosterone, the major active glucocorticoid in rodents, was significantly elevated in acrolein-exposed male rats, whereas females, displaying higher basal corticosterone, showed no acrolein effect (Fig. 2B). The corticosteroid metabolite 11-dehydrocorticosterone was also increased in acrolein-exposed males (Fig. 2C). Moreover, 20α-Dihydroprogesterone, a metabolite of progesterone produced from the catalysis action of AKR enzymes^35^, trended upward in acrolein-exposed females, and was markedly higher in females compared to males at baseline (Fig. 2D). 20α-Dihydroprogesterone was 63-fold higher in air-exposed females relative to air-exposed males and 73-fold higher in acrolein-exposed females relative to acrolein-exposed males (Fig. 2D).

### Acute acrolein exposure increases FFA metabolite levels

Numerous FFA metabolites were increased in acrolein-exposed males, with very few concordant changes observed in acrolein-exposed females (Table 2). Acrolein-exposed males showed consistent increases in saturated and non-saturated hydroxy fatty acids ranging in length from C8 to C27 (Table 2). Several carnitines, such as acetylcarnitine (C2) and deoxycarnitine, were higher in male but not female serum following acrolein exposure (Table 2). Long-chain polyunsaturated fatty acids (LCPUFA) tetradecadienoate (14:2) and hexadecatrienoate (16:3n3) were higher in both sexes following acrolein exposure (males > females). However, males showed further increases in serum omega-3 and omega-6 LCPUFA (i.e., linoleate [alpha or gamma; (18:3n3 or6)] and linoleate (18:2n6)) (Table 2). Interestingly, many FFA metabolites were found at significantly lower levels in air-control females compared to control males (Table 2). Moreover, phospholipids, encompassing the largest lipid profile family, were impacted by acrolein. Multiple triacylglycerol hydrolysis products belonging to monoacylglycerol (MAGs, i.e., 2-oleoylglycerol (18:1)) and diacylglycerol (DAGs, i.e., diacylglycerol (16:1/18:2 [2], 16:0/18:3 [1])) pathways were significantly or near-significantly increased in acrolein-exposed males (Table 2). Further, males also responded to acrolein exposure with higher sphingomyelin metabolites, as well as 3-hydroxybutyrate (3-BHBA), a ketone product. Although there were sex differences at baseline for many phospholipids, none were significantly impacted by acrolein inhalation in females (Table 2).

**Table 2:**
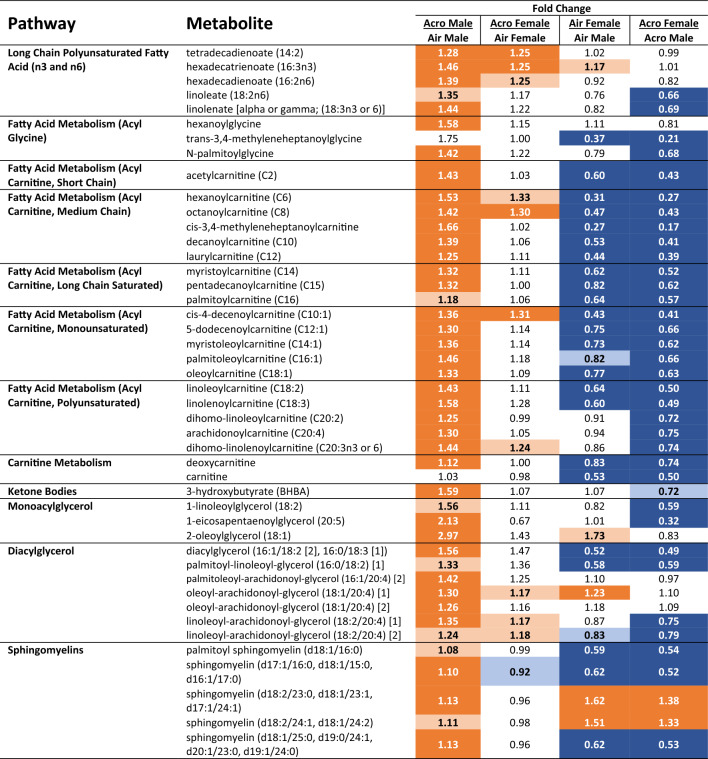
Sex-specific influence of acrolein on alterations in lipid and fatty acid metabolism pathways.

Relative fold difference between air- or acrolein-exposed males or females are shown for each metabolite within pathway. Dark orange and dark blue *p* $\le$ 0.05; light orange and light blue *p* = 0.05–0.10. Orange indicates increased and blue indicates decreased metabolite levels.

### Acrolein changes acetylated catabolic metabolites in male serum

Several branched-chain amino acids (BCAA) were more abundant in males following acrolein exposure (Table 3). Data indicated acrolein-induced changes in males in parent amino acid groups leucine, isoleucine, lysine, as well as a few of their acetylated catabolic metabolites (Table 3). Keto acids (oxo acids, i.e., 4-methyl-2-oxopentanoate and 3-methyl-2-oxobutyrate) and numerous modified N-acetylated amino metabolites (i.e., N-acetylvaline and n-acetyllysine) were higher in acrolein-exposed males, which was not observed in acrolein-exposed females (Table 3). Glutamate metabolism was also impacted in males following acrolein exposure, observed through increased N-acetylglutamine, 4-hydroxyglutamate, and carboxyethyl-GABA (Table 3). Changes in acetylated amino acids were accompanied by altered urea cycle metabolites in acrolein-exposed males, including lower urea and higher ornithine, citrulline, and creatinine metabolites (Table 3). Interestingly, polyamine (N(1) + N(8))-acetylspermidine and its subsequent conversion product spermine was increased in males exposed to acrolein (Table 3). Few amino acid metabolites, 2-pyrrolidinone and putrescine, were uniquely impacted in acrolein-exposed females (Table 3). Male and female air controls displayed numerous significantly different basal serum metabolite levels in a similar manner to sex effects observed in the lipid super-pathway (Table 3).

**Table 3:**
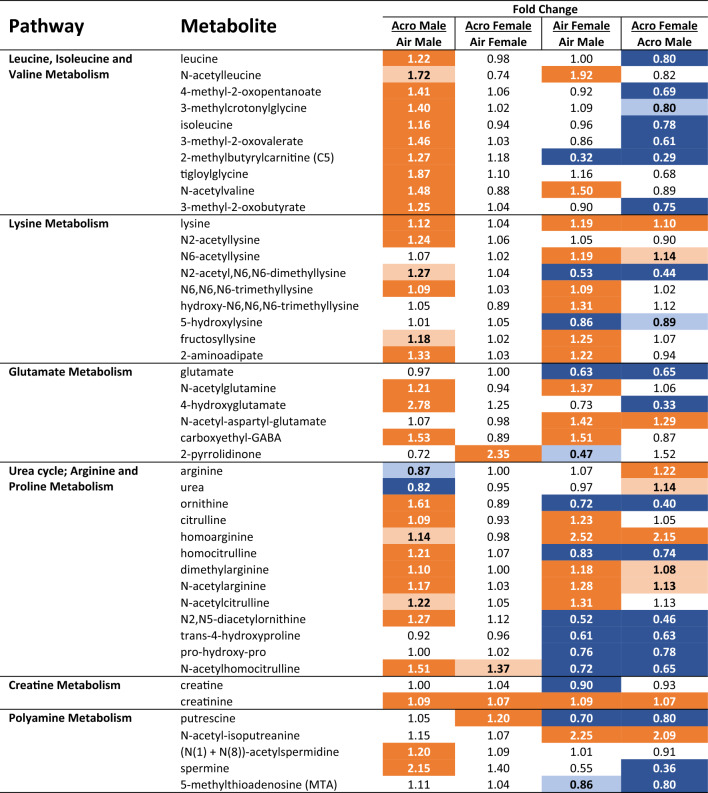
Acrolein-induced perturbations in branched-chain amino acid and nitrogen balance metabolism in males but not females.

Relative fold difference between air- or acrolein-exposed males or females are shown for each metabolite within pathway. Dark orange and dark blue *p* $\le$ 0.05; light orange and light blue *p* = 0.05–0.10. Orange indicates increased and blue indicates decreased metabolite levels.

### Acrolein exposure alters nitrogen balance and redox homeostasis

**Figure 3:**
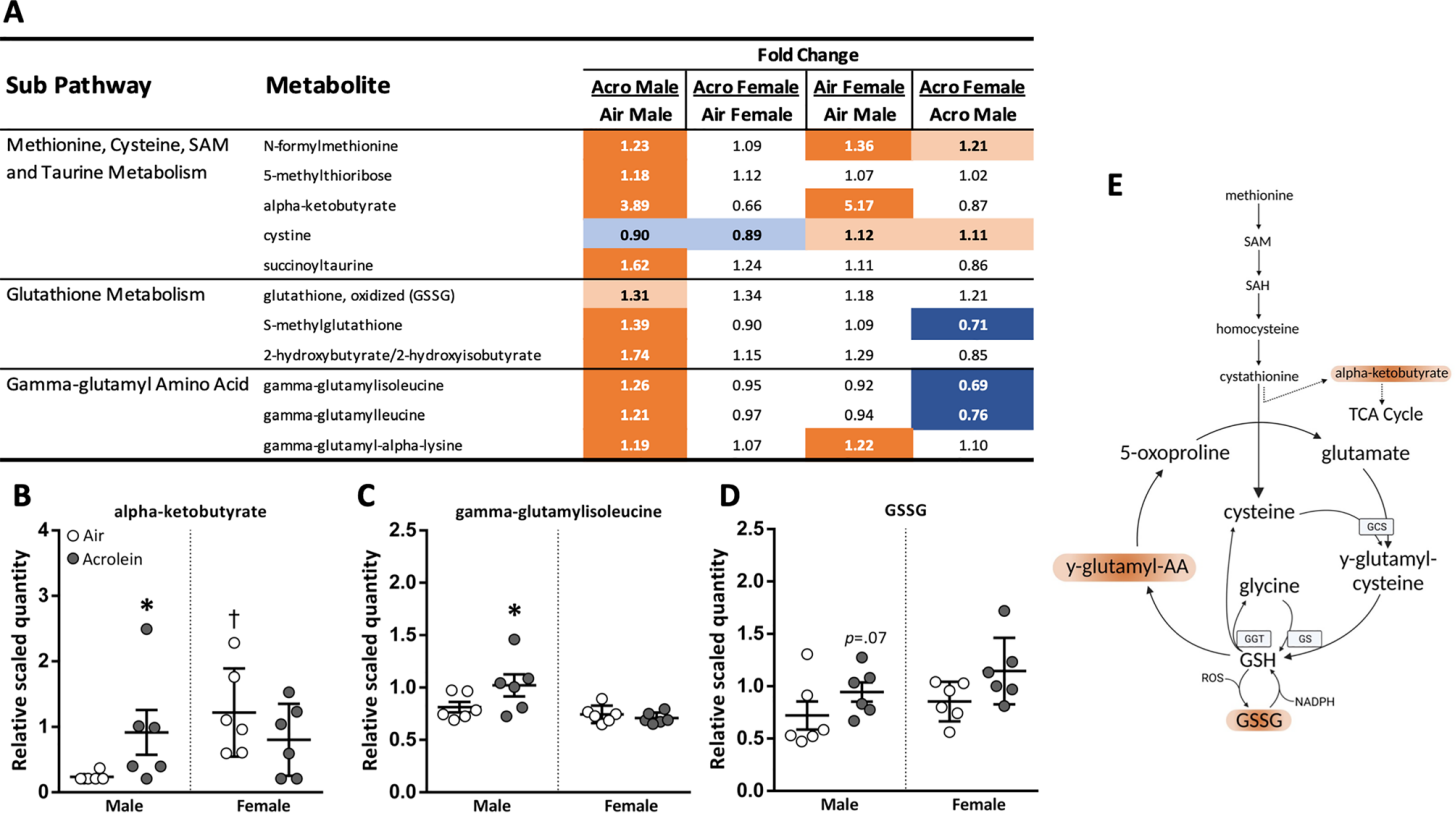
Acrolein-induced changes in oxidative stress metabolism observed through methionine, glutathione, and gamma-glutamyl amino acid pathways. (**A**) depicts heatmap table of metabolites for each sub-pathway, expressed as fold change (relative quantity). Dark orange and dark blue are *p* $\le$ 0.05; light orange and light blue are *p* = 0.05–0.10. Metabolites alpha-ketobutyrate (**B**), gamma-glutamylisoleucine (**C**), and GSSG (**D**) are shown in scatter dot plots (shown as relative scaled quantity; mean ± SEM, *n* = 6/group/sex) to highlight schema of cellular anti-oxidant cycles (**E**).

Acrolein exposure resulted in consistent increases in multiple metabolites related to altered nitrogen and redox homeostasis (Fig. 3). Methionine, cysteine, and taurine metabolites, including N-formylmethionine and succinoyltaurine, were higher in serum of acrolein-exposed males (Fig. 3A). Oxidative-stress related metabolite alpha-ketobutyrate was significantly higher in air females relative to air males and was increased by acrolein exposure in male rats (Fig. 3B). Glutathione (GSH) antioxidant responses to acrolein in males were suggested through significant or near-significant increases in GSH disulfide (GSSG) and multiple gamma-glutamyl amino acids (i.e., gamma-glutamylisoleucine) (Fig. 3A,C,D).

### Microbiome-related biochemicals respond to acute acrolein inhalation in male rats

**Figure 4:**
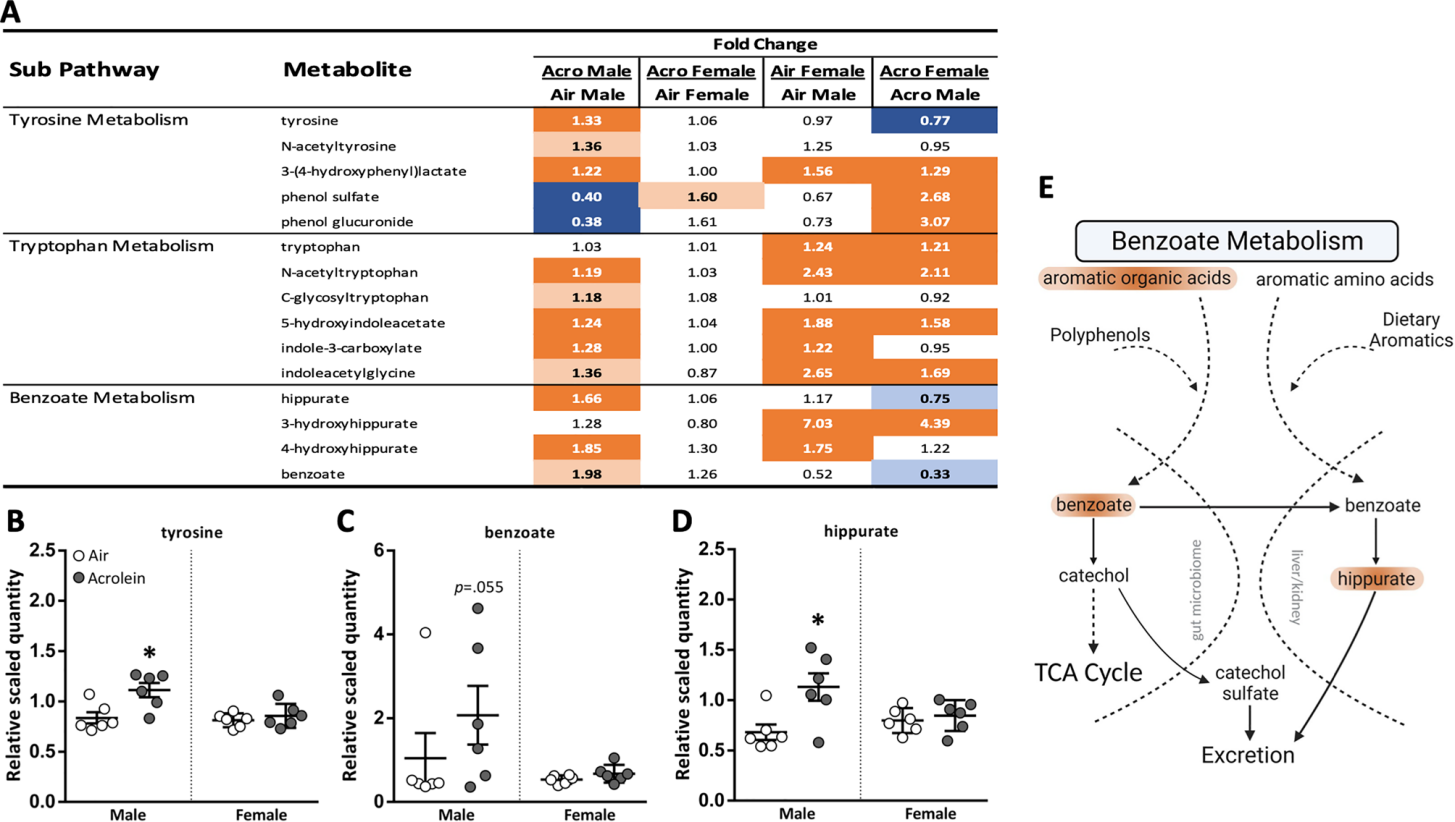
Serum metabolites reflective of enteric microbiome interactions impacted in acrolein-exposed rats. (**A**) depicts heatmap table of metabolites for each sub-pathway, expressed as fold change. Dark orange and dark blue are *p* $\le$ 0.05; light orange and light blue are *p* = 0.05–0.10. Metabolites tyrosine (**B**), benzoate (**C**), and hippurate (**D**) are shown in scatter dot plots (shown as relative scaled quantity; mean ± SEM, *n* = 6/group/sex) to highlight schema of microbiome-related metabolites (**E**).

A number of biochemical changes suggested acrolein altered metabolites linked to gut microbiome (Fig. 4). Tyrosine and its metabolite 3-(4-hydroxyphenyl) lactate were significantly elevated in the serum of acrolein-exposed males (Fig. 4A and B). Acrolein-related perturbations in tryptophan metabolism were noted through elevated levels of nitrogenous tryptophan metabolites (Fig. 4A). Benzoate metabolites, which are carboxylic acids produced from enteric degradation of aromatic compounds, were also increased in males following acrolein inhalation (i.e., benzoate, hippurate, and 4-hydroxyhippurate) (Fig. 4A,C,D). This was further supported by increases in other aromatic organic purine and pyrimidine metabolites, which are also related to changes in nucleotide metabolism (Supplemental Table 1). These metabolites were unaltered in females, albeit several microbiome-associated biochemicals were higher in air control female rats relative to male rats (Fig. 4A).

### Acute acrolein inhalation-induced changes in liver metabolic homeostasis

**Figure 5:**
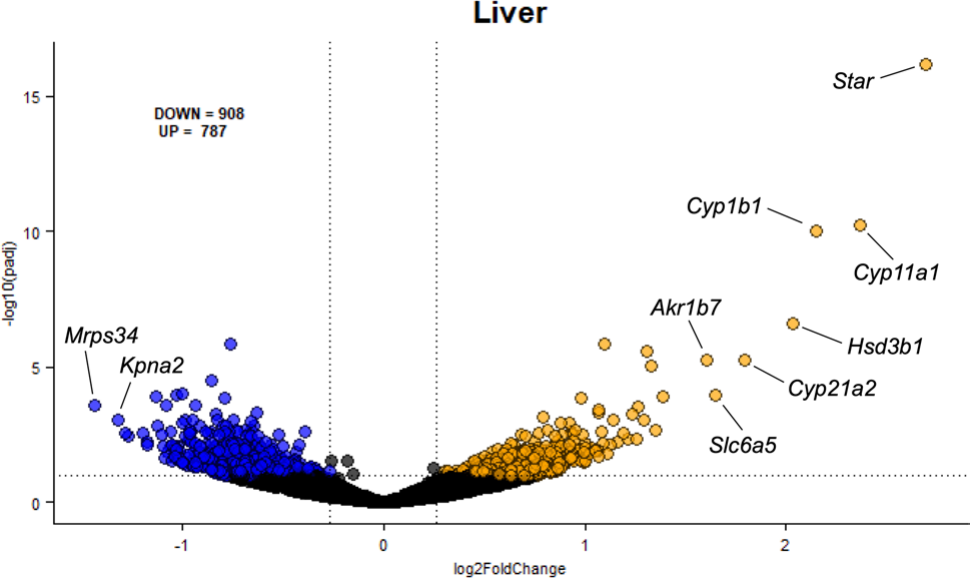
Volcano plot of differentially expressed genes (DEGs) in liver of male rats. Following 4 h air or acrolein exposure, hepatic RNAseq was performed in males. Plot shows − log10(padj) (adjusted *p*-value $\le$ 0.10) and Log2FoldChange (absolute fold change in expression > 20%) for all genes. 1695 DEGs were identified in the liver (787 up- and 908 down-regulated). Blue dots represent significantly down-regulated DEGs and orange dots represent significantly up-regulated DEGs. Prominent differentially expressed genes (by Log2FoldChange) are labeled on the plot.

Due to consistent changes in serum biochemicals within lipid, amino acid, and cholesterol pathways in males but not in females, we performed global gene expression analysis in the liver of males to assess the hepatic contribution to acrolein-induced systemic metabolic impairment. RNAseq analysis revealed 1695 differentially expressed genes (DEGs) (787 up-regulated and 908 down-regulated) in the liver following acrolein exposure (Fig. 5). The one hundred most increased and one hundred most decreased transcripts (by Log2FC) are shown in Supplemental Table 2. Top up-regulated DEGs were related to proteins involved in cell signaling and cytochrome P450 (CYP450) enzymes involved in steroidogenesis, including increased expression of *Star*, *Cyp11a1*, *Cyp1b1*, and *Hsd3b1*, *Cyp21a1*, and *Slc6a5*, as well as AKR encoding *Akr1b7*, involved in acrolein detoxification (Supplemental Table 2)^36^. IPA canonical pathway analysis revealed acrolein-induced mRNA expression changes were mainly related to metabolic deregulation, among other inflammatory and cellular stress signaling changes (Supplemental Table 3). The top predicted canonical pathway altered was Sirtuin signaling, which regulates gluconeogenesis, insulin sensitivity, and lipid metabolism^37^, followed by altered farnesoid X receptor (FXR) activity, which plays a role in maintaining bile acid, glucose, and lipid homeostasis (Supplemental Table 3)^38^. Upstream regulators predicted to mediate observed hepatic mRNA expression changes are found in Supplemental Table 4. Interestingly, insulin and Nrf2 (NFE2L2) regulation were predicted to be down-regulated (Supplemental Table 4).

We utilized the Panther GO-Slim tool to align mRNA expression changes with broad ontological biological processes. GO-Slim analysis indicated over-representation of several catabolic and anabolic pathways in the liver following acrolein exposure (Table 4). Of the 1695 DEGs, 261 were related to primary metabolism. Numerous over-represented process changes pertained to protein, lipid, and carbohydrate metabolism, as well as cellular processes such as endomembrane and Golgi organization (Table 4).

**Table 4:** Functional biological processes impacted in the liver of acrolein-exposed males identified through Panther GO-Slim Biological Process tool using hepatic mRNA expression data.

PANTHER GO-slim biological process	Current genes	Expected genes	Over ( +) under ( −)	Fold enrichment	FDR
Golgi organization (GO:0007030)	9	1.74	+	5.17	1.34E − 02
Organic hydroxy compound biosynthetic process (GO:1901617)	9	1.88	+	4.79	1.91E − 02
Monocarboxylic acid metabolic process (GO:0032787)	16	5.87	+	2.72	2.93E − 02
Endomembrane system organization (GO:0010256)	16	5.98	+	2.68	3.31E − 02
Lipid biosynthetic process (GO:0008610)	19	7.58	+	2.51	2.76E − 02
Ubiquitin-dependent protein catabolic process (GO:0006511)	24	10.21	+	2.35	1.87E − 02
Carbohydrate derivative biosynthetic process (GO:1901137)	22	9.7	+	2.27	4.87E − 02
Modification-dependent protein catabolic process (GO:0019941)	24	10.65	+	2.25	2.88E − 02
Proteolysis involved in cellular protein catabolic process (GO:0051603)	27	12.09	+	2.23	1.92E − 02
Cellular protein catabolic process (GO:0044257)	27	12.12	+	2.23	1.83E − 02
Modification-dependent macromolecule catabolic process (GO:0043632)	24	10.82	+	2.22	3.09E − 02
Protein catabolic process (GO:0030163)	28	13.04	+	2.15	2.36E − 02
Cellular macromolecule catabolic process (GO:0044265)	34	16.22	+	2.1	1.25E − 02
Lipid metabolic process (GO:0006629)	32	15.71	+	2.04	2.11E − 02
Macromolecule catabolic process (GO:0009057)	35	17.79	+	1.97	2.05E − 02
Cellular catabolic process (GO:0044248)	54	27.9	+	1.94	2.06E − 03
Catabolic process (GO:0009056)	61	31.62	+	1.93	7.15E − 04
Organic substance catabolic process (GO:1901575)	51	26.67	+	1.91	3.35E − 03
Proteolysis (GO:0006508)	45	24.99	+	1.8	2.14E − 02
Cellular protein metabolic process (GO:0044267)	96	63.75	+	1.51	9.98E − 03
Organonitrogen compound metabolic process (GO:1901564)	135	92.26	+	1.46	1.95E − 03
Protein metabolic process (GO:0019538)	109	75.39	+	1.45	1.30E − 02
Biosynthetic process (GO:0009058)	135	93.38	+	1.45	2.70E − 03
Primary metabolic process (GO:0044238)	261	181.13	+	1.44	2.63E − 07
Organic substance biosynthetic process (GO:1901576)	133	93.21	+	1.43	4.99E − 03
Cellular biosynthetic process (GO:0044249)	130	91.68	+	1.42	6.94E − 03
Cellular metabolic process (GO:0044237)	261	183.9	+	1.42	6.58E − 07
Cellular macromolecule metabolic process (GO:0044260)	184	130.6	+	1.41	4.66E − 04
Organic substance metabolic process (GO:0071704)	273	197.01	+	1.39	1.37E − 06
Nitrogen compound metabolic process (GO:0006807)	237	172.02	+	1.38	3.81E − 05

The first column shows the top 30 gene ontology (GO) terms and accession identifiers. Subsequent columns show current versus expected genes, direction and fold enrichment (all top 30 GO processes were over-represented (+)), and false discovery rate (FDR).

**Figure 6:**
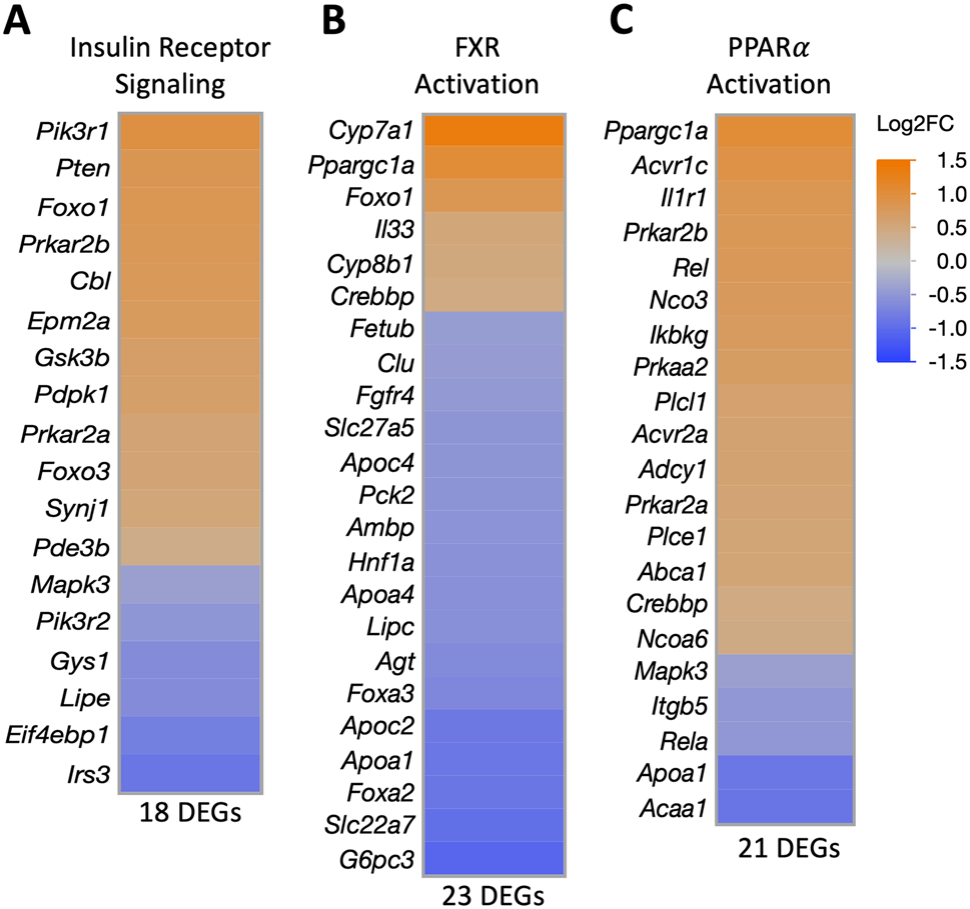
Heatmaps for metabolic pathways impacted by acrolein in male rats. Liver mRNA expression data imported into Ingenuity Pathway Analysis (IPA) identified transcriptional changes in signaling pathways related to insulin receptor function (**A**), farnesoid X receptor (FXR) activation (**B**), and peroxisome proliferator-activated receptor α (PPARα) activation (**C**). Heatmaps of differentially expressed genes (adj. *p*- value $\le$ 0.10) are expressed as Log2FC; orange is up- and blue is down-regulated.

We further assessed overlap of our liver gene expression changes with small molecule biological functions using IPA disease and function tool. Analysis indicated a marked activation of bile acid synthesis, as well as hepatic accumulation of glucose, phospholipids, and fatty acids (Table 5). Interestingly, mRNA changes indicated genes regulating membrane lipid metabolism, sterol metabolism, and lipolysis were downregulated in the liver following acrolein exposure (Table 5). Decreased insulin receptor signaling was observed through 18 DEGs, which included increased *Pi3kr1* and decreased *Irs3* expression (Fig. 6A). FXR activity (23 DEGs) was impacted in acrolein-exposed rats (Fig. 6B). Lastly, we noted acrolein-mediated activation of peroxisome proliferator-activated receptor α (PPARα) pathway in the liver (21 DEGs), which included increased expression of *Ppargc1a* (Fig. 6C). Further information on specific genes involved in insulin receptor signaling, FXR activation, and PPARα activation is in Supplemental Table 5.

**Table 5:**
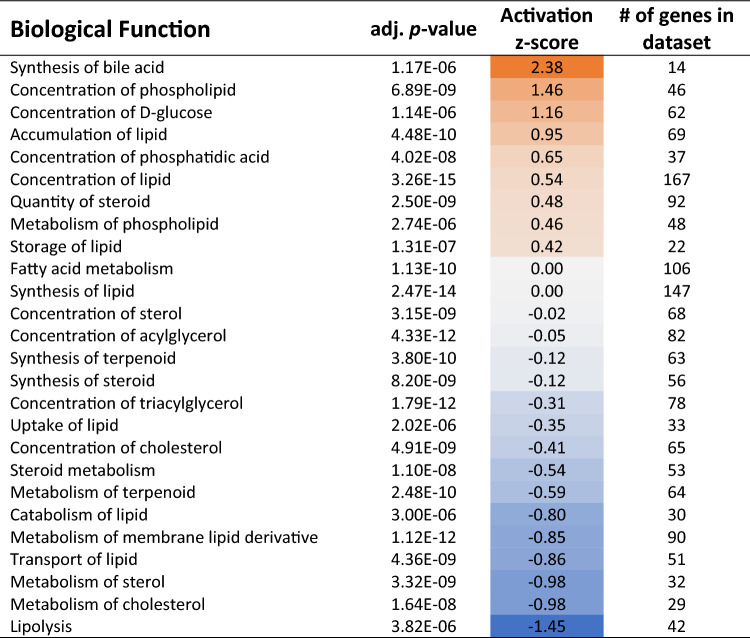
Small molecule processes impacted in the liver identified through Ingenuity Pathway Analysis (IPA) Disease and Functions analysis.

Acrolein-induced liver mRNA expression changes were analyzed in IPA database and implicated in numerous biological functions relating to bile acid synthesis, lipid metabolism, steroid metabolism, cholesterol metabolism, and lipolysis. Adj. *p*-value, activation z-score (orange is up and blue is down), and number of DEGs per pathway are shown.

### Acrolein-induced changes in liver oxidative and cellular stress processes

To further assess acrolein-induced transcriptional changes in other liver homeostatic processes, we examined changes in xenobiotic metabolism, oxidoreductive, and pro-inflammatory pathways. We noted an alteration of acute phase response (APR) signaling (21 DEGs), including an up-regulation of interleukins *Ilr1*, *Il6r*, and *Il33*, but down-regulated *Apoa1*, a cholesterol transport gene (Supplemental Fig. 3A)^39^. Liver results indicated an overall decrease in Nrf2-mediated oxidative stress signaling (23 DEGs), including reduced expression of multiple CYP2 family genes and those involved in oxidative stress (Supplemental Fig. 3B). Acrolein exposure, which increased circulating corticosterone, induced significant changes in hepatic glucocorticoid receptor (GR) signaling (59 DEGs) (Supplemental Fig. 3C). As acrolein also induces sympathetically-mediated catecholamine release in the circulation^29^, the genes involved in adrenergic pathways through cAMP and PI3K/AKT signaling (26 DEGs) were altered (Supplemental Fig. 3D). Acrolein exposure also impacted liver expression of genes pertaining to cell cycle regulation (12 DEGs), autophagy (27 DEGs), mTOR signaling (22 DEGs), and mitochondrial dysfunction (35 DEGs) (Supplemental Fig. 3A–D). Cell cycle regulation changes included increased expression of multiple cyclin kinases (*Cdk6*, *Ccnd2*, and *Ccnh*) (Supplemental Fig. 4A). Hypoxia-induced *Hif1a* expression influencing autophagy and mTOR signaling pathways was increased (Supplemental Fig. 4B and C). Lastly, a change in liver electron transport chain activity was suggested through reduced expression of mitochondrial intermembrane NADH dehydrogenase subunits (*Ndufa13*, *Ndufa8*, *Ndufs7*, *Ndufs3*, *Ndufa2*, and *Ndufv1*) (Supplemental Fig. 4D).

## Discussion

Acrolein is a prominently studied aldehyde known to cause respiratory injury and inflammation that contribute to chronic airway disease^26^. However, recent findings suggest inhaled acrolein may also have systemic health effects^29,30^. Risk assessment for inhaled hazardous air pollutants, including acrolein, does not currently incorporate systemic stress alterations arising from homeostatic neuroendocrine mechanisms, suggesting metabolic markers of disease may be overlooked, especially with chronic exposures. In this study, our goal was to characterize the systemic metabolomic response to acutely inhaled acrolein as a proxy for multi-organ homeostatic disruption and to determine whether sex modified these effects. Male and female rats exhibited baseline metabolomic profile differences. Acute acrolein inhalation induced derangement of multiple catabolic and anabolic processes involving carbohydrate, lipid, steroidogenic, and protein metabolism primarily in male rats, which were associated with 18-fold increase in major acrolein detoxification by-product, 3-HPMA. Interestingly, females, which in comparison to males showed small metabolomic changes, had 33-fold increase in 3-HPMA. Because the liver plays a central role in responding to stress and restoring homeostasis, we performed global transcriptomic profiling in the liver of male rats. Liver mRNA assessment corroborated acrolein-induced shifts in metabolic homeostasis, indicating transcriptomic changes in metabolic and cellular stress signaling pathways. Collectively, acute acrolein exposure induced widespread and sex-specific systemic homeostatic metabolic alterations, which can be linked to neuroendocrine mediated increases in catecholamines and steroidal hormones.

A number of laboratory animal studies have demonstrated that air pollutants induce neural and systemic effects, likely through neuroendocrine activation, and stimulate release of adrenal hormones, which can act as circulating modulators of glucose, lipid, and amino acid metabolism in peripheral tissues^40,41^. We observed extensive baseline metabolomic profile differences across various phospholipids, fatty acids, and amino acid metabolites, including nitrogen and gut microbiome-related precursors, which may underlie the male versus female rat response to acute acrolein inhalation^42^. The overall male-specific response is in line with previously shown neuroendocrine effects of acrolein, where male WKY rats generally displayed enhanced respiratory and adrenal hormone response^30^. It is important to note that although estrous cycle phase is critical in mediating inflammatory responses induced by inhaled irritants^43^, serum gonadal hormones measured in females indicated no significant differences in estrous stage among air- and acrolein-exposed female experimental groups (Supplemental Fig. 5). Prior targeted metabolomic analysis in Sprague–Dawley rats has demonstrated intra-sex differences in lipid and amino acid metabolites^44^, and in humans, sex differences in lipid metabolism are also reported^45,46^, supporting sex-specific regulation of energy balance may impact the metabolomic response to inhalation stress.

High urinary 3-HPMA, an acrolein-specific biomarker observed in cigarette smokers exposed to high levels of acrolein over a long period of time^47^, is associated with adverse metabolic phenotypes, such as increased HOMA-IR, an indicator of insulin resistance^31^. Here, the fold increase of circulating 3-HPMA in acrolein-exposed female rats was nearly double that of acrolein-exposed males. Considering 3-HPMA is produced by phase II detoxification of acrolein through GSH and AKR enzymes^48^, a large sex-specific increase in 3-HPMA indicates greater acrolein detoxification in females when compared to males. This finding suggests sex-specific detoxification capacity may, in part, underlie differential metabolomics responses in acrolein-exposed males versus females. Higher baseline 20α-dihydroprogesterone in female rats, a metabolite formed by AKR enzymatic activity^35^, supports differences in basal AKR activity. Liver mRNA expression data in males indicated marked up-regulation of aldehyde metabolizing AKR gene *Akr1b7*, which is also inducible by nuclear receptor FXR activity to maintain glucose and lipid homeostasis, as well as increase bile acid metabolism^49,50^. It is important to note that inhaled acrolein is deposited mainly in the nasal airways and will not translocate systemically as a parent compound. Thus, it is presumably metabolized within nasal airways, which heavily express a variety of chemical metabolizing enzymes^51^. Acrolein effects on the nasal Nrf2-mediated activation of AKR and GSH conjugation pathways might partly underlie sex-specific differences in acrolein-induced metabolic alterations^52,53^. Lower capacity of metabolic detoxification in males when compared to females might increase their susceptibility to long-term acrolein exposure-induced health consequences.

Male-specific acute acrolein inhalation-induced increases in cholesterol and glucocorticoid metabolites support the contribution of SAM and HPA processes in mediating the metabolomic effects observed in males. We have previously shown increases in plasma corticosterone in male WKY rats following acrolein inhalation under the same exposure paradigm, which was accompanied by other markers of systemic stress such as lymphopenia^30^. Glucocorticoids and catecholamines released in response to ozone-induced stress modify metabolic processes in liver, adipose tissue, and muscle^22,40^. Broadly, this pattern of adrenal activity may underly sex-specific acrolein-induced outcomes noted across our serum metabolomics results following acrolein exposure. Given that the lack of serum biochemical changes in female rats occurred in tandem with an absence of stress-mediated corticosterone synthesis, acrolein inhalation-induced increases in circulating stress hormones likely mediate multi-organ metabolic changes. It is noteworthy that in air-exposed female rats, the baseline corticosterone levels were significantly higher than those of air-exposed males. These baseline differences could underlie some of the basal sex differences in these classes of metabolites.

Circulating catecholamines released after neuroendocrine activation act upon β-adrenergic receptors of adipocytes to liberate fatty acids and glycerol into circulation in response to shifting energy requirements^54,55^. Serum metabolomics has shown male WKY rats exposed to 1 ppm ozone for 6 h have higher acylcarnitine and lysolipid metabolites^15^, with similar results recapitulated in humans exposed to 0.3 ppm ozone for 2 h^16^. Here, we show a 4 h exposure to 3.16 ppm acrolein caused hyperglycemia and increased serum fatty acid and triglyceride catabolic products (MAGs and DAGs) in males, implying acrolein-induced nasal injury prompted sympathetic outflow to enhance adipose lipolysis. Acylcarnitine-conjugated fatty acids are typically transported to the liver and undergo beta-oxidation into acetyl-CoA for mitochondrial respiration^56^. During a surplus of oxidation substrates, the liver may form incomplete acetyl-CoA products, or ketones^56^. Considering males showed elevated ketone bodies (3-BHBA) in response to acute acrolein inhalation, results suggest discordant levels between adipose lipolysis and hepatic fatty acid oxidation. Excessive lipolysis has detrimental impacts on peripheral tissues through ectopic lipid accumulation, which may lead to hepatic steatosis^53^. Although the potential outcomes of current lipidomic changes are likely short lived and will need to be addressed by further long-term exposures studies, our findings indicate acute acrolein inhalation disrupts lipid metabolism in male rats.

Acute acrolein-induced increases in circulating BCAA may reflect stress-induced protein catabolism. Under metabolic stress, BCAA are transformed into branched-chain ketoacids (oxo acids) via branched-chain aminotransferase, which predominantly occurs in muscle tissue^57^. Given increased serum ketoacid metabolites in males, acrolein-induced stress likely augments BCAA homeostasis and possible protein utilization in other organs such as the liver. The liver does not oxidize BCAA, but rather utilizes precursor amino acids for synthesis of glycolytic intermediates and acute phase reactants^57,58^. Further, under conditions of heightened circulating amino acids, urea is generated to excrete nitrogenous waste and maintain nitrogen balance^59^. Although urea was not impacted by acrolein exposure in either sex, urea cycle intermediates ornithine and citrulline were higher in serum of acrolein-exposed males, indicating an activation of this process.

Systemic inflammation and oxidative stress are suggested to play a pivotal role in the pathogenesis of metabolic disorders, including diabetes^11^. Higher gamma-glutamyl amino acid and GSH metabolites may support a utilization of increased circulating BCAA for redox homeostasis cycles. Studies using PM exposures, which deposits along the entire respiratory tract, associate a release of lung pro-inflammatory mediators with systemic inflammation^60,61^. However, although increases in GSH disulfide levels suggests systemic oxidative stress during short acrolein exposure, systemic inflammation might remain inhibited by released corticosterone, evidenced by lymphopenia after acrolein and ozone exposure^30,62^.

The enteric microbiome plays a complex role in physiological reactions to environmental stressors, where gut-microbial derived metabolites enter circulation and act as messengers in peripheral organs^63^. Prior studies using ozone exposures in mice show changes in the gut microbiome that are suggested to impact airway hyperresponsiveness^64,65^. Interestingly, the current results suggest acute acrolein inhalation alters microbiome-related benzoate and hippurate metabolites in males, which is consistent with prior observances in male WKY rats that show acute ozone-induced changes in gut microbiome-related metabolites^15^. Multiple increased aromatic compounds are derived from microbial breakdown of dietary components. Considering rats were fasted during exposure and sample collection (~ 4-6 h), these components were likely mobilized in response to perceived stress from acrolein inhalation. The impact of these metabolites in respiratory or systemic responses to acrolein are yet to be determined.

Overall, acute acrolein-induced changes in liver transcriptional response were reflective of stress-induced increases in the serum adrenal-derived stress hormones^30^ and metabolomic profile, likely as a result of adipose lipolysis and muscle protein catabolism in males. Molecular pathways changed in the liver suggest a transcriptional response to lipid, cholesterol, and glucose accumulation. Decreased expression of genes involved in hepatic lipolysis supports the notion fatty acids in circulation did not originate from the liver but are a result of adipocyte-liberated fatty acids shuttling to the liver for processing. Unlike the inhibition of genes involved in bile acid metabolism observed following ozone exposure^15^, acrolein inhalation induced genes involved in hepatic bile acid synthesis. Hepatic bile acid metabolism is the chief pathway for maintenance of cholesterol homeostasis, and impaired bile acid synthesis over time causes dyslipidemia and fatty liver disease^66^. Bile acids are also known to act as FXR nuclear receptor ligands to regulate glucose and lipid homeostasis^67^, and liver top canonical pathway alterations indicated altered FXR and GPCR-related signaling. These results suggest the liver may have initiated counteractive measures in response to increased circulating cholesterol and fatty acid substrates.

Liver transcriptional data collected from male rats suggested acute acrolein inhalation-induced impairment of insulin signaling. Increased *Pi3kr1* expression, observed in acrolein-exposed male rats, inhibits insulin sensitivity by halting insulin-stimulated cellular glucose transport^68^. Further, acute acrolein inhalation also enhanced expression of genes relating to PPARα function, such as *Ppargc1a*, which are induced by glucocorticoids to modulate fatty acid oxidation, mitochondrial biogenesis, and gluconeogenesis^69^. Prior experiments in male WKY rats have associated acute ozone exposure with an inhibition of glucose-mediated insulin release^15^, and male Fisher-344 rats exposed to ozone show glucocorticoid-dependent alterations in functionally-similar liver insulin signaling genes^22^. Alterations of these key metabolic signaling pathways suggest the ability of inhaled acrolein to impact hepatic mitochondrial function and insulin signaling, which might contribute to an increased risk for developing adverse metabolic phenotypes over continued exposure.

Lastly, acute acrolein inhalation-induced changes in liver expression were not limited to metabolic processes, but also indicated involvement of inflammatory and cellular stress signaling, correlating with results of previous ozone studies^15,19^. Long-term exposure to air pollutants, particularly PM, is linked with development of liver disease, such as NAFLD^70,71^. Pollutants which usually contain high levels of acrolein, such as cigarette smoke, have also been suggested to impact NAFLD progression^72^. Here, an acute acrolein exposure induced marked expression changes of mediators involved in inflammatory processes, as well as glucocorticoid and PI3K/AKT signaling. Interestingly, we observed decreased hepatic expression of NADH oxidoreductase genes, which may impair GSH reductase activity and allow for the cellular accumulation of GSSG^73^, concordant with GSSG increase in serum. Although these outcomes are reversible under acute exposure conditions, results support the notion that long-term acrolein might impact oxidoreductive and pro-inflammatory processes in the liver.

It is important to note the limitations of this acute exploratory study, particularly with regards to the implications of these findings associated with long-term exposure to acrolein. While serum metabolomics is a widely used method to generate a broad overview of the host response as a diagnostic tool for disease^74^, the exact source and cause of changes in circulating biochemicals can only be speculated and may be influenced by unknown factors. Current hypotheses generated from serum metabolomics and liver transcriptomics represent acute neuroendocrine effects which may shift with exposure cessation or continuation and cannot inform long-term outcomes, especially across species. Female liver mRNA expression profiling may also reveal biological underpinnings of the current sex-specific metabolic response to acrolein. However, our observational results are consistent with evidence that suggests air pollution is linked with metabolic stress and highlight the need to consider metabolic outcomes associated with long-term acrolein inhalation. Future intervention studies should investigate tissue- and receptor-specific functions in mediating these effects, which may indicate targets for intervention. Lastly, the highest acrolein concentration used in this study is not representative of ambient acrolein pollution levels (3.6–10.7 ppb)^75–77^. However, acrolein inhalation through cigarette smoke can result in high exposure spikes to comparable or greater levels (i.e., > 3.16 ppm)^78^. Differences in metabolic susceptibility have been noted in rodent models, where Goto-Kakizaki rats, a T2DM model strain, showed worsened metabolic outcomes following acrolein inhalation^29^, suggesting lower levels may pose an increased risk for those with pre-existing conditions.

In conclusion, we show that a single acute exposure to acrolein, a nasal irritant, induces sex-specific systemic metabolic derangement, observed through shifts in glucose, lipid, and protein metabolism in male rats. Acrolein-induced significant metabolic derangement in males but only modest effects in females was associated with higher levels of acrolein detoxification byproduct in the female serum when compared to males. Increases in serum FFA and BCAA indicate neuroendocrine-mediated effects on adipose lipolysis and muscle protein catabolism, which can impact energetic and inflammatory processes in the liver. The sex-specific increases in male adrenal corticosterone metabolites further highlights the involvement of neuroendocrine mechanisms in orchestrating multi-organ stress reactions following acrolein exposure. Changes in the male liver transcriptome corroborated responses in cholesterol, fatty acid, and protein metabolism, and suggest the liver as a target for circulating metabolite and stress hormones, albeit the long-term implications of these findings in health outcomes like hepatic insulin resistance and NAFLD require further investigation. Nevertheless, this sexually dimorphic systemic metabolomic response to acute acrolein inhalation may explain the susceptibility to metabolic disruption in males.

## Materials and methods

### Animals

Data presented here were obtained from samples collected in our prior acrolein exposure study^30^. Male and female WKY rats were purchased from Charles River Laboratories (Raleigh, NC). Rats were housed in the AAALAC accredited US EPA Office of Research and Development Research Triangle Park animal facility until 12–13 weeks of age and at atmospheric housing conditions of 21 °C ± 1 °C, 50–65% relative humidity under a 12 h light/dark cycle. Pair-housed rats (sexes housed separately) were kept in polycarbonate cages containing hardwood chip bedding and Enviro-Dri^®^ enrichment wrinkle paper for enrichment and were provided Purina (5001) rat chow (Brentwood, MO) and water provided ad libitum (except during exposure). At exposure, males weighed ~ 250–300 g and females weighed ~ 150–200 g. All experimental procedures presented here received prior approval from the US EPA Health Institutional Animal Care and Use Committee.

### Experimental design

Experimental design details are provided in our prior publication^30^. Briefly, rats were randomly assigned to either filtered air (control) (*n* = 8/sex) or acrolein (*n* = 8/sex) exposure groups based on body weight via an Excel macro (male and female rats randomized separately). Over 1–2 days prior to exposure, rats were acclimated to nose-only inhalation tubes. On day 1, rats underwent two 2 h acclimations, separated by 4 h, and on the second day, rats underwent a single 4 h acclimation. On exposure day, veterinary-grade artificial tears (Akorn Animal Health, Inc., Lake Forest, IL) were applied to the eyes and rats were exposed via nose only inhalation to air (0 ppm) or acrolein (3.16 ppm) for 4 h. To be able to assess concentration-dependent effects of acrolein on respiratory parameters using head-out plethysmography in real-time, as explained in our prior study^30^, the first 30 min of acrolein exposure included increasing half-log concentrations (0, 0.10, 0.316, and 1 ppm acrolein) lasting 7.5 min/concentration. Immediately following exposure, rats were necropsied for sample collection (all necropsies completed within 2 h after end of exposure; sexes exposed and necropsied separately). All experimental methods were performed in accordance with relevant guidelines and regulations. This study is reported in accordance with Animal Research: Reporting of In Vivo Experiments (ARRIVE) guidelines.

### Acrolein exposures

Acrolein exposures were conducted as described previously^29,30^. Briefly, acrolein was obtained as a gas (Airgas, Morrisville, NC). Desired concentrations were generated for exposure using modified in-house gas blending system, which was modulated by mass flow controllers (MKS, Andover, MA) that meter flow of test agent and dilution air (medical grade air), and were directed to a 52-outlet port nose-only inhalation tower at a flow rate of 0.35L/min/port. Acrolein concentration delivery to nose-only exposure towers were monitored using and Agilent 6890 gas chromatography analyzer with flame ionization detector and a 624 capillary column (Supelco, Bellefonte, PA). Flows were adjusted as needed and no deviations in target concentrations were noted. Atmospheric conditions of temperature (21.8 °C ± 0.3 °C) and relative humidity (38.9% ± 2.7%) were monitored in exposure room once per hour during exposure.

### Necropsy and sample collection

Immediately after exposure, animals were weighed and euthanized via an intraperitoneal injection of sodium pentobarbital (> 200 mg/kg) (Fatal-plus; Covetrus, Portland, ME) in a staggered manner between air- and acrolein-exposed rats. Blood samples were collected from the abdominal aorta using a vacutainer serum separator tube. Blood samples were centrifuged at 3500 *rpm* at 4 °C for 10 min and then serum was stored at − 80 °C for later analysis. During blood collection, blood glucose levels were measured via a Bayer Contour glucometer and test strips. Then, uniform liver sections were extracted, flash frozen in liquid nitrogen, and stored at − 80 °C for RNAseq.

### Assessment of serum FFA

Serum FFA were measured using kits purchased from Cell Biolabs (Cell Biolabs, Inc., San Diego, CA). Kit protocols were followed as directed by manufacturer and modified for use on the Konelab Arena 30 clinical analyzer (Thermo LabSystems, Espoo, Finland).

### Serum metabolomics

Serum samples stored at − 80 °C were selected for global serum metabolic profiling to be performed by Metabolon, Inc. (Durham, NC) (*n* = 6/group/sex). Detailed experimental methods for serum metabolomic assessment involving sample preparation, ultra-high performance liquid chromatography-tandem mass spectrometry (UHPLC-MS/MS), and small-molecule identification/quantification are outlined in our prior publications^16,79^ and others^80,81^. Briefly, samples were prepared by the automated MicroLab STAR® system (Hamilton Company, Reno, NV). Prior to extraction, several recovery standards were added for quality control (QC). To remove protein, dissociate small molecules bound to protein or trapped in the precipitated protein matrix, and to recover chemically diverse metabolites, proteins were precipitated with methanol under vigorous shaking for 2 min (Glen Mills GenoGrinder 2000) followed by centrifugation. Aliquots of the resulting extract were separated into five fractions: two for analysis by two separate reverse phase (RP)/UHPLC-MS/MS methods with positive ion mode electrospray ionization (ESI), one for analysis by RP/UHPLC-MS/MS with negative ion mode ESI, one for analysis by HILIC/UHPLC-MS/MS with negative ion mode ESI, and one sample was reserved for backup. Samples were placed briefly on a TurboVap® (Zymark) to remove the organic solvent. The sample extracts were stored overnight under nitrogen before preparation for analysis.

Several QC samples were assessed in tandem with experimental samples. A pooled matrix sample created from a small volume of each experimental sample served as a technical replicate throughout the data set. Extracted water samples served as process blanks and a mixture of carefully chosen QC standards, which do not interfere with the measurement of endogenous compounds, were spiked into every analyzed sample, allowing instrument performance monitoring and aiding chromatographic alignment. Instrument variability was determined by calculating the median relative standard deviation (RSD) for the standards that were added to each sample prior to injection into the mass spectrometers. Overall process variability was determined by calculating the median RSD for all endogenous metabolites (i.e., non-instrument standards) present in 100% of the pooled matrix samples. Median RSD for internal standards was 3% and for endogenous biochemicals was 8%, satisfying Metabolon’s acceptance criteria.

### Ultrahigh performance liquid chromatography-tandem mass spectroscopy

All methods utilized a Waters ACQUITY UHPLC and a Thermo Scientific Q-Exactive high resolution/accurate mass spectrometer interfaced with a heated electrospray ionization (HESI-II) source and Orbitrap mass analyzer operated at 35,000 mass resolution. The dehydrated extract was then reconstituted in solvents compatible to each of the four methods. Each reconstitution solvent contained a series of standards at fixed concentrations to ensure injection and chromatographic consistency. One aliquot was analyzed using acidic positive ion conditions, chromatographically optimized for more hydrophilic compounds. In this method, the extract was gradient eluted from a C18 column (Waters UPLC BEH C18-2.1 × 100 mm, 1.7 µm) using water and methanol, containing 0.05% perfluoropentanoic acid (PFPA) and 0.1% formic acid (FA). Another aliquot was also analyzed using acidic positive ion conditions. However, it was chromatographically optimized for more hydrophobic compounds. In this method, the extract was gradient eluted from the same aforementioned C18 column using methanol, acetonitrile, water, 0.05% PFPA and 0.01% FA and was operated at an overall higher organic content. Another aliquot was analyzed using basic negative ion optimized conditions using a separate dedicated C18 column. The basic extracts were gradient eluted from the column using methanol and water, with 6.5 mM ammonium bicarbonate at pH 8. The fourth aliquot was analyzed via negative ionization following elution from a HILIC column (Waters UPLC BEH Amide 2.1 × 150 mm, 1.7 µm) using a gradient consisting of water and acetonitrile with 10 mM ammonium formate, pH 10.8. The MS analysis alternated between MS and data-dependent MS^n^ scans using dynamic exclusion. The scan range generally covered 70–1000 m/z.

### Data extraction and compound identification and curation

Metabolon Inc. hardware and software processed raw data and identified peaks with QC. Compound identification was completed by comparison with a previously curated library of known entries, purified standards, or recurring unknown entities. Metabolon maintains a library based on authenticated standards that contains the retention time/index (RI), mass to charge ratio (*m/z)*, and chromatographic data (including MS/MS spectral data) on all molecules present in the library. Furthermore, biochemical identifications are based on three criteria: retention index within a narrow RI window of the proposed identification, accurate mass match to the library +/− 10 ppm, and the MS/MS forward and reverse scores between the experimental data and authentic standards. The MS/MS scores are based on a comparison of the ions present in the experimental spectrum to the ions present in the library spectrum. Utilizing all three points of data can be used to distinguish and differentiate unique biochemical signatures across a catalog of more than 3300 commercially available purified standard compounds registered in the Metabolon Laboratory Information Management System for determination of analytical characteristics. Additional mass spectral entries have been created for structurally unnamed biochemicals, which have been identified by virtue of their recurrent nature (both chromatographic and mass spectral). The QC and curation processes were designed to ensure accurate and consistent identification of true chemical entities, and to remove those representing system artifacts, mis-assignments, and background noise. Library matches for each compound were checked for each sample and corrected if necessary.

### Metabolite normalization, statistics, and bioinformatics

Peaks obtained from MS were quantified using trapezoidal method of area-under-the-curve. For runs in a study spanning multiple days, a batch normalization step was performed to correct for variation resulting from inter-day instrumental differences. Thus, each compound underwent a “block correction”, where samples were corrected in run-day blocks by registering the medians to equal 1.00 and normalizing each data point proportionately. Standard statistical analyses are performed in ArrayStudio/Jupyter Notebook on log transformed data. Following log transformation and imputation of missing metabolite values, if any, with the minimum observed value for each compound, analysis of variance (ANOVA) contrasts was performed to identify biochemical differences between groups. Analysis by two-way ANOVA identified biochemicals exhibiting significant interaction and main effects for experimental parameters of exposure condition (acrolein or air), sex, and interaction. Significantly altered metabolites were determined at *p* $\le$ 0.05 and were considered trending towards significance if *p* = 0.05–0.10. The false discovery rate (FDR) (*q*-value) was calculated. However, ANOVA outcomes are reported as un-adjusted (Supplemental Table 1). Table 1 outlines serum metabolite statistical comparisons summary, showing ANOVA results by exposure and sex.

PCA, which uses an orthogonal transformation to highlight patterns within the dataset, was performed to assess metabolite profile clustering by sex and exposure condition. For each identified biochemical, metabolomics platform (Metabolon, Inc.) assigned knowledge-based pathway annotations. Annotations were drawn from the Kyoto Encyclopedia of Genes and Genomes (KEGG) pathways, which link molecular changes to larger conserved pathways^82^, and the Human Metabolome Database (HMDB), which is a comprehensive database of known human metabolites^83^. KEGG and HMDB annotations for metabolites quantified in this study are found in Supplemental Table 1. Figures featuring metabolomics results were generated using GraphPad Prism v9 and BioRender (www.biorender.com, accessed 2023).

### RNA isolation and mRNA sequencing

Uniform portions of frozen liver (10–20 mg) (males only; *n* = 5–6) were sectioned for RNA extraction and processed with RNeasy non-fibrous tissue mini kits (Qiagen, Valencia, CA) following manufacturer directions. RNA quantity and purity (260/230 and 260/280 ratios) were spectrophotometrically quantified using a Nanodrop 1000 (ThermoFisher Scientific Inc., Waltham, MA). RNA integrity was assessed by the RNA 6000 LabChip® kit and a 2100 Bioanalyzer (Agilent Technologies, Santa Clara, CA) using a RIN cutoff of 7. Sample processing for mRNA sequencing was performed on the Apollo324 automated system (Takara Bio Inc., Kusatsu, Japan) for library prep with PrepX mRNA 48 protocol v19, using the PrepX™ RNA-seq for Illumina Library Kit (Takara), SuperScript III reverse transcriptase (ThermoFisher), and AMPure XP Beads (Agilent). PCR amplification with 48 index primers was run for 16 cycles and resulting PCR product quality was analyzed again via Qubit (ThermoFisher) and bioanalyzer. An RNA-seq library was then prepared with Wafergen’s PrepX mRNA 48 protocol and dsDNA products were prepared from cDNA. Each library was sequenced according to Illumina NextSeq 500, with a final concentration of 2.2 pM + 2% PhiX and run for 75 cycles (Illumina Inc., San Diego, CA).

### mRNA sequencing data normalization, statistics, and bioinformatics

Sequenced mRNA reads were mapped to the rat genome (rn7) using ensemble release 105 (Partek Flow suite, R version 4.1.3), resulting in 24,091 identified genes. Genes with fewer than 1 read in 10 out of 11 samples were removed, leaving 20,580 genes. An average of 15.5 million reads were mapped per sample with a standard deviation of 5.5 million. PCA was performed to identify any samples that did not cluster and should be removed, and two liver samples failed to cluster by tissue. After removal of those tissues, remaining samples had 15.8 million reads per sample with a standard deviation of 5.3 million, with an average read per gene of 766. DEGs were assessed in DESeq2 package in R (v1.34.0)^84^ and normalized reads were assessed for acrolein effects. Gene expression was considered significant if the fold change (FC) was greater than 1.2 (upregulation) or less than 0.833 (downregulation) and the Benjamini–Hochberg FDR $\le$ 0.1. Liver RNAseq array data are found at National Center for Biotechnology Information Gene Expression Omnibus (NCBI GEO: GSE247698). RNAseq plot generation and bioinformatics was conducted using shrunken Log2FoldChanges (DESeq2 shrinkage method) to improve accuracy of DEG analysis.

Ingenuity Pathway Analysis (IPA) software (QIAGEN, Redwood City, CA; www.qiagen.com/ingenuity; last accessed June 22, 2023) was used to compare our mRNA expression data with previously reported databases, thus identifying significant trends through overlaps in expression changes. IPA was used to identify major canonical pathways and signaling network alterations, and upstream analysis tool in IPA was used to predict the involvement of various upstream regulators; an |activation z-score| $\ge$ 2 was set for meaningful prediction of regulator involvement in transcriptional changes^85^. Another feature of IPA, disease and functions analysis, yielded identification of acrolein-induced liver mRNA changes related to functions of small molecular pathway changes in the liver. Log2FC of genes were used to create heatmaps in JMP Pro statistical software (v17, SAS, Cary, NC), where orange is increased expression and blue is decreased expression. Finally, we utilized Panther GO-Slim Biological Processes (PANTHER 17.0 online software, http://www.pantherdb.org/; last accessed June 22, 2023), which is a core biodata resource to determine overlap of our DEGs with a curated databased of ~ 3000 gene ontology (GO) Slim terms of an annotated database pertaining to biological processes (Benjamini–Hochberg FDR $\le$ 0.1)^86^.

### General statistics

Analysis of blood glucose and serum FFA were performed using GraphPad Prism v9. Data were assessed for outliers using a robust regression outlier test (ROUT, Q = 1%; none were found). To ensure assumptions of normality and homoscedasticity within an analysis of variance (ANOVA) were met, data underwent Shapiro-Wilks normality (α = 0.05) and Levene’s equality of variance tests and were transformed (Y = Log(Y)) as needed. Data were analyzed with a two-way ANOVA (exposure, sex) and Holm-Sidak post-hoc. Endpoints were considered significant when *p* $\le$ 0.05.

### Disclosure statement

The research described in this article has been reviewed by the Center for Public Health and Environmental Assessment, U.S. EPA and approved for publication. Approval does not signify that the contents necessarily reflect the views and the policies of the Agency, nor does mention of trade names of commercial products constitute endorsement or recommendation for use.

### Supplementary Information

Supplementary Information.

Supplementary Tables.

## Data Availability

Datasets generated or analyzed during the current study may be made available upon reasonable request by contacting the corresponding author, Dr. Urmila Kodavanti.

## Abbreviations

3-BHBA: 3-Hydroxybutyrate
3-HPMA: 3-Hydroxypropyl mercapturic acid
AKR: Aldo–keto reductase
ANOVA: Analysis of variance
APR: Acute phase response
BCAA: Branched-chain amino acids
CYP450: Cytochrome P450
DAG: Diacylglycerol
DEGs: Differentially expressed genes
ESI: Electrospray ionization
FC: Fold change
FDR: False discovery rate
FFA: Free fatty acids
FXR: Farnesoid X receptor
GO: Gene ontology
GSH: Glutathione
HMDB: Human metabolome database
HPA: Hypothalamic–pituitary–adrenal
IPA: Ingenuity pathway analysis
KEGG: Kyoto encyclopedia of genes and genomes
LCPUFA: Long-chain polyunsaturated fatty acids
MAG: Monoacylglycerol
NAFLD: Non-alcoholic fatty liver disease
PCA: Principal component analysis
PFPA: Perfluoropentanoic acid
PPARα: Peroxisome proliferator-activated receptor α
QC: Quality control
RI: Retention index
RP: Reverse phase
RSD: Relative standard deviation
SAM: Sympathetic-adrenal-medullary
SEM: Standard error of the mean
T2DM: Type 2 diabetes mellitus
UHPLC-MS/MS: Ultra-high performance liquid chromatography-tandem mass spectrometry
WKY: Wistar-Kyoto

## References

[1] Lovic D (2020). The growing epidemic of diabetes mellitus. Curr. Vasc. Pharmacol..

[2] Rooney MR (2023). Global prevalence of prediabetes. Diabetes Care.

[3] World Health Organization (WHO). Diabetes Fact Sheet. *World Health Organization*, Geneva, Switzerland. 2023. https://www.who.int/news-room/fact-sheets/detail/diabetes. Accessed 8 July 2023.

[4] Saeedi P (2019). Global and regional diabetes prevalence estimates for 2019 and projections for 2030 and 2045: Results from the International Diabetes Federation Diabetes Atlas, 9^th^ edition. Diabetes Res. Clin. Pract..

[5] Grundy SM (2005). Diagnosis and management of the metabolic syndrome: An American Heart Association/National Heart, Lung, and Blood Institute Scientific Statement. Circulation.

[6] Landrigan PJ (2018). The Lancet Commission on pollution and health. Lancet.

[7] Health Effects Institute (HEI). State of global air 2020. Special Report. *Health Effects Institute*, Boston, MA. 2020. ISSN 2578–6873 © 2020**.**

[8] Thiering E, Heinrich J (2015). Epidemiology of air pollution and diabetes. Trends Endocrinol. Metab..

[9] Liu F (2023). Exposure to air pollution and prevalence of metabolic syndrome: A nationwide study in China from 2011 to 2015. Sci. Total Environ..

[10] Rajagopalan S, Brook RD (2012). Air pollution and type 2 diabetes: Mechanistic insights. Diabetes.

[11] Grundy SM (2004). Clinical management of metabolic syndrome: Report of the American Heart Association/National Heart, Lung, and Blood Institute/American Diabetes Association conference on scientific issues related to management. Arterioscler. Thromb. Vasc. Biol..

[12] O'Neill MS (2007). Air pollution and inflammation in type 2 diabetes: A mechanism for susceptibility. Occup. Environ. Med..

[13] Shoelson SE, Lee J, Goldfine AB (2006). Inflammation and insulin resistance. J. Clin. Invest..

[14] Della Guardia L, Shin AC (2022). PM_2.5_-induced adipose tissue dysfunction can trigger metabolic disturbances. Trends Endocrinol. Metab..

[15] Miller DB (2015). Inhaled ozone (O3)-induces changes in serum metabolomic and liver transcriptomic profiles in rats. Toxicol. Appl. Pharmacol..

[16] Miller DB (2016). Ozone exposure increases circulating stress hormones and lipid metabolites in humans. Am. J. Respir. Crit. Care Med..

[17] Snow SJ (2021). Diets enriched with coconut, fish, or olive oil modify peripheral metabolic effects of ozone in rats. Toxicol. Appl. Pharmacol..

[18] Jackson TW (2022). Adrenal stress hormone regulation of hepatic homeostatic function after an acute ozone exposure in wistar-kyoto male rats. Toxicol. Sci..

[19] Colonna CH (2021). The role of hepatic vagal tone in ozone-induced metabolic dysfunction in the liver. Toxicol. Sci..

[20] Hodge MX, Henriquez AR, Kodavanti UP (2021). Adrenergic and glucocorticoid receptors in the pulmonary health effects of air pollution. Toxics.

[21] Kodavanti UP (2019). Susceptibility variations in air pollution health effects: Incorporating neuroendocrine activation. Toxicol. Pathol..

[22] Rose M, Filiatreault A, Williams A, Guénette J, Thomson EM (2023). Modulation of insulin signaling pathway genes by ozone inhalation and the role of glucocorticoids: A multi-tissue analysis. Toxicol. Appl. Pharmacol..

[23] US EPA (2022). Initial List of Hazardous Air Pollutants with Modifications.

[24] US EPA (2017). 2017 AirToxScreen: Assessment Results.

[25] Haussmann HJ (2012). Use of hazard indices for a theoretical evaluation of cigarette smoke composition. Chem. Res. Toxicol..

[26] Moghe A (2015). Molecular mechanisms of acrolein toxicity: Relevance to human disease. Toxicol. Sci..

[27] Alarie Y (1973). Sensory irritation by airborne chemicals. CRC Crit. Rev. Toxicol..

[28] Hazari MS (2014). A single exposure to acrolein desensitizes baroreflex responsiveness and increases cardiac arrhythmias in normotensive and hypertensive rats. Cardiovasc. Toxicol..

[29] Snow SJ (2017). Respiratory effects and systemic stress response following acute acrolein inhalation in rats. Toxicol. Sci..

[30] Alewel DI (2023). Sex-specific respiratory and systemic endocrine effects of acute acrolein and trichloroethylene inhalation. Toxicol. Lett..

[31] Feroe AG, Attanasio R, Scinicariello F (2016). Acrolein metabolites, diabetes and insulin resistance. Environ. Res..

[32] Wang B (2023). Acrolein exposure impaired glucose homeostasis and increased risk of type 2 diabetes: An urban adult population-based cohort study with repeated measures. Environ. Sci. Technol..

[33] Feng X (2022). Urinary acrolein metabolites, systemic inflammation, and blood lipids: Results from the National Health and Nutrition Examination Survey. Chemosphere.

[34] Henriquez AR (2022). Stress drivers of glucose dynamics during ozone exposure measured using radiotelemetry in rats. Environ. Health Perspect..

[35] Penning TM (2015). The aldo-keto reductases (AKRs): Overview. Chem. Biol. Interact..

[36] Singh M, Kapoor A, Bhatnagar A (2015). Oxidative and reductive metabolism of lipid-peroxidation derived carbonyls. Chem. Biol. Interact..

[37] Wu QJ (2022). The sirtuin family in health and disease. Signal. Transduct. Target Ther..

[38] Sun L, Cai J, Gonzalez FJ (2021). The role of farnesoid X receptor in metabolic diseases, and gastrointestinal and liver cancer. Nat. Rev. Gastroenterol. Hepatol..

[39] Cochran BJ, Ong KL, Manandhar B, Rye KA (2021). APOA1: A protein with multiple therapeutic functions. Curr. Atheroscler. Rep..

[40] Snow SJ, Henriquez AR, Costa DL, Kodavanti UP (2018). Neuroendocrine regulation of air pollution health effects: Emerging insights. Toxicol. Sci..

[41] Seematter G, Binnert C, Martin JL, Tappy L (2004). Relationship between stress, inflammation and metabolism. Curr. Opin. Clin. Nutr. Metab. Care.

[42] Krumsiek J (2015). Gender-specific pathway differences in the human serum metabolome. Metabolomics..

[43] Fuentes N, Cabello N, Nicoleau M, Chroneos ZC, Silveyra P (2019). Modulation of the lung inflammatory response to ozone by the estrous cycle. Physiol. Rep..

[44] Ruoppolo M (2018). Targeted metabolomic profiling in rat tissues reveals sex differences. Sci. Rep..

[45] Blaak E (2001). Gender differences in fat metabolism. Curr. Opin. Clin. Nutr. Metab. Care.

[46] Wang X, Magkos F, Mittendorfer B (2011). Sex differences in lipid and lipoprotein metabolism: It's not just about sex hormones. J. Clin. Endocrinol. Metab..

[47] Carmella SG (2007). Quantitation of acrolein-derived (3-hydroxypropyl)mercapturic acid in human urine by liquid chromatography-atmospheric pressure chemical ionization tandem mass spectrometry: Effects of cigarette smoking. Chem. Res. Toxicol..

[48] Stevens JF, Maier CS (2008). Acrolein: Sources, metabolism, and biomolecular interactions relevant to human health and disease. Mol. Nutr. Food Res..

[49] Ge X (2011). Aldo-keto reductase 1B7 is a target gene of FXR and regulates lipid and glucose homeostasis. J. Lipid Res..

[50] Schmidt DR (2011). AKR1B7 is induced by the farnesoid X receptor and metabolizes bile acids. J. Biol. Chem..

[51] Dahl AR, Hadley WM (1991). Nasal cavity enzymes involved in xenobiotic metabolism: Effects on the toxicity of inhalants. Crit. Rev. Toxicol..

[52] Penning TM (2017). Aldo-keto reductase regulation by the Nrf2 system: Implications for stress response, chemotherapy drug resistance, and carcinogenesis. Chem. Res. Toxicol..

[53] Alewel DI (2023). Differential transcriptomic alterations in nasal versus lung tissue of acrolein-exposed rats. Front. Toxicol..

[54] Carey GB (1998). Mechanisms regulating adipocyte lipolysis. Adv. Exp. Med. Biol..

[55] Nonogaki K (2000). New insights into sympathetic regulation of glucose and fat metabolism. Diabetologia.

[56] Nguyen P (2008). Liver lipid metabolism. J. Anim. Physiol. Anim. Nutr. (Berl.).

[57] Mattick JSA, Kamisoglu K, Ierapetritou MG, Androulakis IP, Berthiaume F (2013). Branched-chain amino acid supplementation: Impact on signaling and relevance to critical illness. Wiley Interdiscip. Rev. Syst. Biol. Med..

[58] Chandel NS (2021). Amino acid metabolism. Cold Spring Harb. Perspect. Biol..

[59] Rafoth RJ, Onstad GR (1975). Urea synthesis after oral protein ingestion in man. J. Clin. Invest..

[60] van Eeden SF, Hogg JC (2002). Systemic inflammatory response induced by particulate matter air pollution: The importance of bone-marrow stimulation. J. Toxicol. Environ. Health A.

[61] Finnerty K (2007). Instillation of coarse ash particulate matter and lipopolysaccharide produces a systemic inflammatory response in mice. J. Toxicol. Environ. Health A.

[62] Henriquez AR (2021). The dynamicity of acute ozone-induced systemic leukocyte trafficking and adrenal-derived stress hormones. Toxicology.

[63] Cryan JF (2019). The microbiota-gut-brain axis. Physiol. Rev..

[64] Cho Y (2018). The microbiome regulates pulmonary responses to ozone in mice. Am. J. Respir. Cell Mol. Biol..

[65] Cho Y (2019). Sex differences in pulmonary responses to ozone in mice. Role of the microbiome. Am. J. Respir. Cell Mol. Biol..

[66] Chiang JY (2013). Bile acid metabolism and signaling. Compr. Physiol..

[67] Chiang JYL, Ferrell JM (2020). Bile acid receptors FXR and TGR5 signaling in fatty liver diseases and therapy. Am. J. Physiol. Gastrointest. Liver Physiol..

[68] Li M (2022). Trends in insulin resistance: Insights into mechanisms and therapeutic strategy. Signal. Transduct. Target Ther..

[69] van Raalte DH, Li M, Pritchard PH, Wasan KM (2004). Peroxisome proliferator-activated receptor (PPAR)-alpha: A pharmacological target with a promising future. Pharm. Res..

[70] Chen J (2021). The influence of PM_2.5_ exposure on non-alcoholic fatty liver disease. Life Sci..

[71] Tarantino G, Capone D, Finelli C (2013). Exposure to ambient air particulate matter and non-alcoholic fatty liver disease. World J. Gastroenterol..

[72] Lin C, Rountree CB, Methratta S, LaRusso S, Kunselman AR, Spanier AJ (2014). Secondhand tobacco exposure is associated with nonalcoholic fatty liver disease in children. Environ. Res..

[73] Wu J, Jin Z, Zheng H, Yan LJ (2016). Sources and implications of NADH/NAD(+) redox imbalance in diabetes and its complications. Diabetes Metab. Syndr. Obes..

[74] Zhang A, Sun H, Wang X (2012). Serum metabolomics as a novel diagnostic approach for disease: A systematic review. Anal. Bioanal. Chem..

[75] De Woskin R, Greenberg M, Pepelko W, Strickland J (2003). Toxicological Review of Acrolein; in Support of Summary Information on the Integrated Risk Information System (Iris).

[76] Alwis KU, de Castro BR, Morrow JC, Blount BC (2015). Acrolein exposure in U.S. tobacco smokers and non-tobacco users: NHANES 2005–2006. Environ. Health Perspect..

[77] deCastro BR (2014). Acrolein and asthma attack prevalence in a representative sample of the United States adult population 2000–2009. PLoS One.

[78] Pauwels CGGM (2018). Cigarette filter ventilation and smoking protocol influence aldehyde smoke yields. Chem. Res. Toxicol..

[79] Snow SJ (2020). Offspring susceptibility to metabolic alterations due to maternal high-fat diet and the impact of inhaled ozone used as a stressor. Sci. Rep..

[80] Dehaven CD, Evans AM, Dai H, Lawton KA (2010). Organization of GC/MS and LC/MS metabolomics data into chemical libraries. J. Chem. Inform..

[81] Evans AM, DeHaven CD, Barrett T, Mitchell M, Milgram E (2009). Integrated, nontargeted ultrahigh performance liquid chromatography/electrospray ionization tandem mass spectrometry platform for the identification and relative quantification of the small-molecule complement of biological systems. Anal. Chem..

[82] Kanehisa M, Goto S (2000). KEGG: Kyoto encyclopedia of genes and genomes. Nucleic Acids Res..

[83] Wishart DS (2007). HMDB: The human metabolome database. Nucleic Acids Res..

[84] Love MI, Huber W, Anders S (2014). Moderated estimation of fold change and dispersion for RNA-seq data with DESeq2. Genome Biol..

[85] Krämer A, Green J, Pollard J, Tugendreich S (2014). Causal analysis approaches in ingenuity pathway analysis. Bioinformatics.

[86] Thomas PD (2022). PANTHER: Making genome-scale phylogenetics accessible to all. Protein Sci..

