# Supplementary material for: Serum metabolome and liver transcriptome reveal acrolein inhalation-induced sex-specific homeostatic dysfunction
Source: Sci Rep. 2023 Dec 1;13:21179. doi: 10.1038/s41598-023-48413-w (PMC10692194; doi:10.1038/s41598-023-48413-w)
Supplement: Supplementary file 1 — Supplementary Information. [file 41598_2023_48413_MOESM1_ESM.docx]

**On-line data supplement**

**Serum metabolome and liver transcriptome reveal acrolein inhalation-induced sex-specific homeostatic dysfunction**

Devin I. Alewel^a^, Katherine M. Rentschler^a^, Thomas W. Jackson^a^, Mette C. Schladweiler^b^, Anna Astriab-Fisher^b^, Paul A. Evansky^b^, and Urmila P. Kodavanti^b^*

^a^Oak Ridge Institute for Science and Education Research Participation Program, U.S. Environmental Protection Agency, Research Triangle Park, NC, United States of America; ^b^Public Health and Integrated Toxicology Division, Center for Public Health and Environmental Assessment, U.S. Environmental Protection Agency, Research Triangle Park, North Carolina, United States of America.

**ORCID IDs:**

Devin I. Alewel: 0000-0003-3415-9477

Thomas W. Jackson: 0000-0002-7996-0412

Urmila P. Kodavanti: 0000-0001-6333-1024

**Running Title:** Acute acrolein inhalation causes sex-specific metabolic stress

***Corresponding Author:**

Dr. Urmila P. Kodavanti, PhD, DABT

Center for Public Health and Environmental Assessment

Public Health and Integrated Toxicology Division

U.S. Environmental Protection Agency

109 T.W. Alexander Dr.

Research Triangle Park, NC 27711 USA

E-mail: [kodavanti.urmila@epa.gov](mailto:kodavanti.urmila@epa.gov)

**Methods**

**Serum neuroendocrine hormone assessment.**

Serum progesterone and follicle-stimulating hormone (FSH) levels were assessed using Milliplex magnetic bead panels (rat-specific) RPTMAG-86K and MSHMAG-21K (Millipore-Sigma, Burling, MA) and carried out following manufacturer guidelines. For each hormone, median fluorescent intensity was measured and analyzed via a 5-parameter logistic curve to calculate sample concentration (corrected for dilution factor) (Luminex Corporation, Austin, TX).

**Neuroendocrine hormone statistics.**

Plasma hormones progesterone and FSH were analyzed using a non-parametric t-test (two-tailed Mann-Whitney test). Results were considered significant when *p* < .05.

**Results**

**Circulating gonadal hormones measured in air- and acrolein-exposed female rats.**

To assess the possible influence of estrous cycling on lack of metabolomic outcomes observed in females, we assessed circulating gonadal hormone levels (Supplemental Figure 4). Serum progesterone and FSH levels were similar among air- and acrolein-exposed females (Supplemental Figure 4A and 4B). Previously, in this same cohort of female rats, we published data on serum estradiol and luteinizing hormone (LH) levels, where we showed that this acrolein-exposure paradigm did not alter female gonadal hormone levels and that air and acrolein groups did not indicate significant differences in estrous stage or basal hormone levels^1^. Taken together, hormones governing estrous cycling suggest no major cycle differences between air and acrolein exposure groups.

**References**

1. Alewel DI, et al. Sex-specific respiratory and systemic endocrine effects of acute acrolein and trichloroethylene inhalation. *Toxicol Lett*. 2023 Jun 1;**382**:22-32. doi: 10.1016/j.toxlet.2023.05.005. PMID: 37201588.

**Supplemental Figure Legends**

**Supplemental Figure 1.** Analysis of circulating clinical chemistry markers showing sex-specific acrolein effects on blood glucose and serum free fatty acids (FFA). Blood glucose (**A**) and serum FFA (**B**) levels were measured in air- or acrolein-exposed male and female rats. Scatter dot plots show all points, mean ± SEM (n= 7-8/group/sex). *Significant acrolein effect within sex at *p* $\leq$ .05.

**Supplemental Figure 2.** Principal component analysis of serum metabolomic results assessed separately in males and females. Air- or acrolein-exposed females are depicted in **A** (n = 6/group/sex) and air- or acrolein-exposed males in **B** (n = 6/group). Male and female rats were exposed to air or acrolein for 4hr and then serum was stored for later metabolomic assessment.

**Supplemental Figure 3.** Heatmaps for inflammatory signaling pathways impacted by acrolein in male rats. Transcriptional changes in multiple immune and hormonal signaling pathways of the liver were impacted by acrolein. Heatmaps show acute phase response signaling (**A**), Nrf2-mediated oxidative stress (**B**), glucocorticoid receptor signaling (**C**), and PI3K/AKT signaling (**D**). Differentially expressed genes are expressed as Log2FC, where orange is up- and blue is down-regulated following acrolein exposure.

**Supplemental Figure 4.** Heatmaps of acrolein-induced changes in cellular processes of the liver. Multiple transcriptional changes were observed in cellular stress pathways of acrolein-exposed male rats. Heatmaps show altered cell cycle regulation (**A**), autophagy (**B**), mTOR signaling (**C**), and mitochondrial dysfunction (**D**). Differentially expressed genes are expressed as Log2FC, where orange is up- and blue is down-regulated following acrolein exposure.

**Supplemental Figure 5.** Serum progesterone and follicle-stimulating hormone (FSH) measured in female rats. Circulating gonadal hormones progesterone (**A**) and FSH (**B**) were assessed in female air- or acrolein-exposed rats. Scatter dot plots show all points, mean ± SEM (n= 6/group).

**Supplemental Figure 1.**

**

**

**Supplemental Figure 2.**

**Supplemental Figure 3.**

**Supplemental Figure 4.**

**Supplemental Figure 5.**
